# Tales from New York City, the pandemic epicenter: A case study of COVID-19 impact on clinical and translational research training at the Icahn School of Medicine at Mount Sinai

**DOI:** 10.1017/cts.2020.560

**Published:** 2020-11-23

**Authors:** Layla Fattah, Inga Peter, Keith Sigel, Janice L. Gabrilove

**Affiliations:** 1 ConduITS, the Institute for Translational Sciences, Icahn School of Medicine, New York, NY, USA; 2 Department of Genetics and Genomic Sciences, Icahn School of Medicine at Mount Sinai, New York, NY, USA; 3 Department of Medicine, Icahn School of Medicine at Mount Sinai, New York, NY, USA; 4 Clinical Research Education Program, Graduate School of Biomedical Sciences, Icahn School of Medicine at Mount Sinai, New York, NY, USA; 5 Tisch Cancer Institute, Icahn School of Medicine at Mount Sinai, New York, NY, USA

**Keywords:** Postdoctoral trainees, professional development, COVID-19

## Abstract

In the spring of 2020, New York City was at the epicenter of the COVID-19 pandemic in the USA, resulting in disruption of TL1 and KL2-mentored Clinical and Translational Science (CTS) research at the Icahn School of Medicine at Mount Sinai (ISMMS). The impact of the pandemic on trainees’ research productivity and career plans was explored using a qualitative survey. Participant responses were analyzed using coding and categorization. Six key themes emerged: redirection of effort, reduced access to people, lack of access to resources, home as a workplace, future uncertainty, and stress and anxiety. Insight into participant experiences allows for the development of support strategies and resources to address trainee needs.

## Introduction

In the spring of 2020, New York City was at the epicenter of the COVID-19 pandemic in the USA. This has had a devastating and disruptive impact on the landscape of graduate education and training in academic health systems [1,2]. At its peak in early April, with over 6300 new cases and 590 deaths per day in New York City [3], major teaching hospitals underwent unprecedented changes to manage the enormous surge in COVID-19 patients. Education, research, and clinical care environments changed dramatically due to pandemic response efforts. On March 17 2020, as the COVID-19 cases surged, the Icahn School of Medicine at Mount Sinai (ISMMS) made the decision to suspend human subject research projects for all non-COVID-19-related research studies. This was a necessary decision that nevertheless had broad implications for the implementation and conduct of research studies and research training. As we moved past the peak of cases for the Mount Sinai Health System (MSHS), we were compelled to examine the impact this pandemic had on our 2019–2020-funded cohort of mentored Clinical and Translational Science (CTS) research trainees and scholars.

The impact of the COVID-19 pandemic on medical student and postgraduate education in the clinical arena has been the focus of attention within healthcare systems and has been explored elsewhere in the literature [1,2,4]. In early April, during the initial wave of the pandemic, the National Center for Advancing Translational Sciences (NCATS) surveyed Clinical and Translational Science Award Program hubs, variably impacted by COVID-19, in an effort to begin to assess the initial impact of the pandemic on training [5]. This coincided with the peak of cases in NYC, making it difficult for ISMMS trainees to find the time to respond, and thus difficult to evaluate the impact at our institution at the COVID-19 epicenter. As a result, there has been less focus on qualitatively evaluating the challenges confronted during this pandemic on CTS training at its epicenter, where the academic enterprise has been profoundly affected. We were, therefore, interested in exploring the impact of the pandemic specifically on the trainee’s research productivity in the short term, and the perceived longer term impact on the trainees’ career plans and progression. Thus, we could potentially identify where professional, practical, and well-being support could be offered to trainees in the short term, and where accommodations might be made to extend periods of training and funding for these individuals. In the longer term, we sought to develop a holistic approach to supporting trainees and developing plans that might prevent similar disruptions in the future to trainees at ISMMS.

For this reason, we chose to survey our 2019–2010 cohort of mentored TL1 predoctoral and postdoctoral trainees as well as KL2 junior faculty scholars, during a period beyond the chaos of the peak caseload in New York City. Surveying the trainees in late May, by which time the cases of COVID-19 in New York City had reduced to roughly 10% of the peak, allowed them the time and opportunity to fully reflect and assess the impact of pandemic-related disruptions on their professional and personal growth as researchers. We sought to better understand trainees’ experiences, in order to determine where and how support and resources could be better provided, going forward. In addition, we hoped to gain insight into unanticipated challenges encountered, so as to best anticipate needs for future trainees.

## Methods

We sought to delineate challenges faced by our 2019–2020 TL1 predoctoral trainees (MD/MSCR candidates) and TL1 postdoctoral trainees (clinical postdoctoral trainees at fellowship, PGY stages 5–7) and KL2 scholars (junior faculty), in order to best inform strategies and resources required to mitigate the impact of this unanticipated crisis on mentored clinical and translational research career development. To accomplish this goal, the faculty worked in conjunction with the education program manager and evaluator to design a short anonymous qualitative survey. The survey questions employed are provided in Table 1.

**Table 1.** COVID-19 impact survey questions

Question 1: As a result of COVID-19, has your job role changed significantly? Yes/No
Please tell us more about this:
Question 2: As a result of COVID-19, have you experienced any new barriers to your training or research? Yes/No
Please tell us more about this:
Question 3: As result of COVID-19, have there been any specific tasks or projects that you have not been able to complete? Yes/No
Please tell us more about this:
Question 4: As a result of COVID-19, have your career plans been impacted? Yes/No
Please tell us more about this:
Question 5: Please tell us about any other impact on your training or career as a result of COVID-19?
Question 6: Do you have any other thoughts or comments you would like to share?

The survey was built in REDCap (a browser-based database and data collection software) and circulated by email to all KL2 scholars (*n* = 4), TL1 postdoctoral trainees (*n* = 3), and TL1 predoctoral Patient-Oriented Research Training and Leadership (PORTAL) trainees (*n* = 6) at ISMMS. Qualitative data responses to questions were analyzed as a single dataset. This qualitative data was coded and categorized. This was conducted manually by initially assigning “codes” through close interrogation of the data. This involved reading and rereading the comments to identify and label recurrent words, themes, and concepts. Data was then denoted into codes, and codes were then grouped into categories. This process was conducted by two of the authors (LF and KS) and codes agreed upon.

## Results

**Fig. 1.**
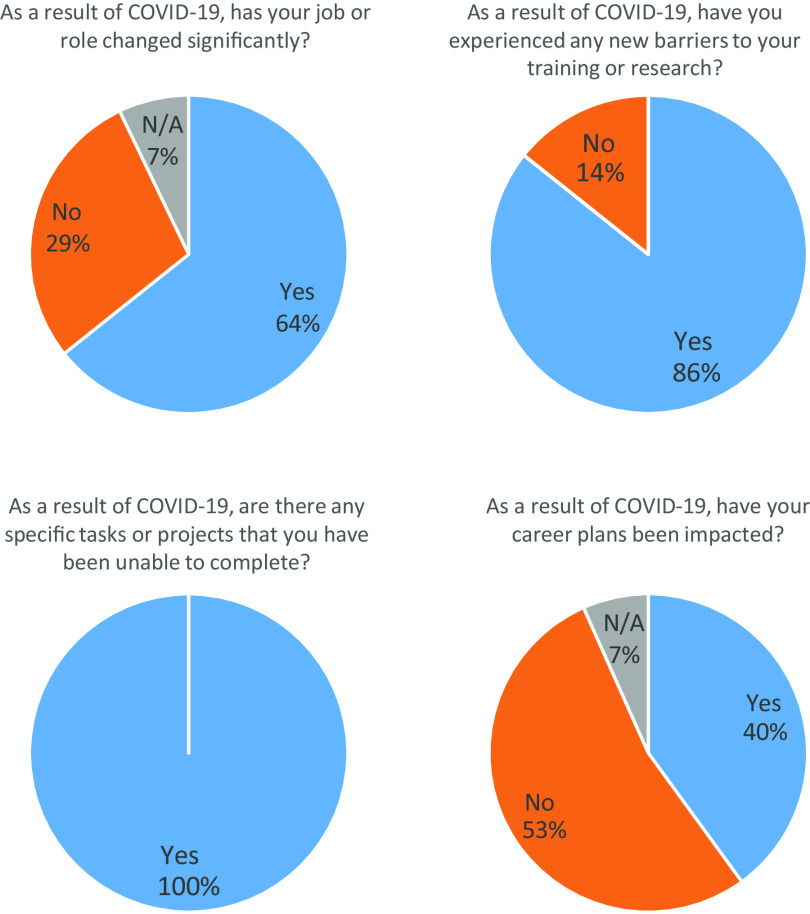
Responses to survey questions (*n* = 13).

We surveyed our cohort of TL1 and KL2 trainees, funded during the time period, 2019–2020. A 100% (*n* = 13) response rate was achieved. As a result of COVID-19, 64% of respondents reported that their jobs or roles had changed significantly (Fig. 1). Eighty-six percent of respondents reported experiencing new barriers to training and/or research (Fig. 1). One-hundred percent of participants reported barriers to complete tasks or projects as a result of COVID-19. In addition, 40% of respondents reported that their career plans had been adversely impacted (Fig. 1).

**Fig. 2.**
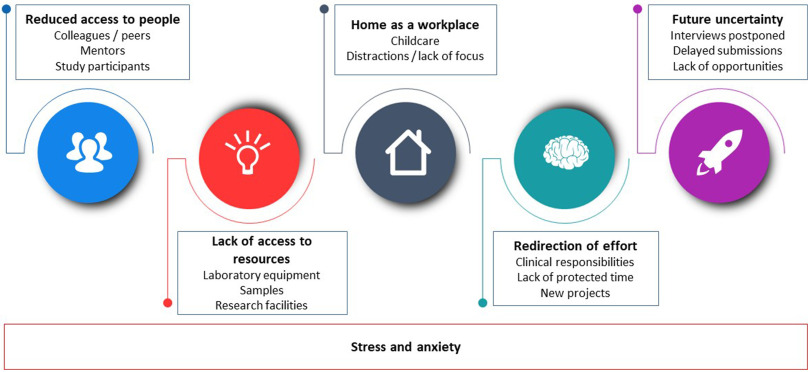
KL2 and TL1 challenges and concerns due to COVID-19.

Analysis of the qualitative survey data gave rise to the identification of six key themes: redirection of effort, reduced access to people, lack of access to resources, home as a workplace, future uncertainty, and stress and anxiety (Fig. 2).

### Redirection of Effort

As a result of the surge in patient numbers at MSHS, the majority of our trainees were redeployed, either to care for COVID-19 patients or to cover colleagues who had taken up this role.

“I had to cover consults and urgent phone calls on behalf of a redeployed fellow.” Participant 4

Trainees who were not permitted to cover clinical duties were asked to redirect their research efforts toward COVID-19-related investigations. The absence of protected time coupled with a significant interruption in focus considerably impaired the ability of trainees to achieve critical milestones and continue to pursue their own research projects.

“Protected research time was eliminated and my schedule was being changed at a moments notice, so I couldn’t really plan research.” Participant 6

### Lack of Access to Resources

As laboratories closed and a stay-at-home order was implemented, participants no longer had access to a host of different research relevant resources. Trainees were no longer able to access supplies, patient samples, or research facilities that they needed to continue with their research. Access to the Mount Sinai Data Warehouse, a clinical data repository for use in clinical and translational research, was challenging and delays in responses to data requests were reported by trainees. Similarly, access to the IRB was also reported to be slow. This lack of access to laboratory and onsite clinical research facilities was a widely reported barrier to both training and research.

“My projects include the usage of cells which require an every day maintenance when in culture. Due to the shutdown I had to completely stop working with those cells.” Participant 7

“Significant delays in data requests.” Participant 3

### Reduced Access to People

Next, reduced access to faculty members, mentors, and team members was reported. As more senior faculty members were redeployed, trainees were no longer able to access the day-to-day support of their mentors.

“…delays in data requests, lack of availability of faculty mentors. Everyone’s focus is on COVID.” Participant 13

In addition, the lack of access to research participants was highlighted by those conducting patient-oriented research studies with patients or patient samples.

“I am unable to get patient samples without in-person clinics in session. And then it will be difficult to process these samples in the future with lab capacity rules.” Participant 4

### Home as a Workplace

The home environment became a challenge for productivity generally. The shutdown of other services across New York City, such as schools and offices, made home life more challenging through increased childcare and schooling commitments.

“Lack of childcare has been a major barrier and will continue to be so.” Participant 7

The unfamiliar environment of home as a workplace provided distractions for both those with and without childcare demands, and created a lack of structure that had a direct impact on productivity.

“It has been very challenging/distracting to operate virtually during this time.” Participant 10

“Being at home is distracting and not an environment I am used to working in. I’m not as productive or efficient as usual.” Participant 8

### Future Uncertainty

Participants expressed concerns regarding their career development and uncertainty about the future due to missed opportunities directly related to COVID-19. As a result of the pandemic, one participant reported having interviews postponed, and others reported not being able to submit grant applications on time. Several participants expressed concerns regarding a lack of future academic career opportunities as a result of the setbacks experienced through COVID-19.

“Before COVID-19 I was applying for a position; which has a big delay to provide an answer due to the availability of the grant. Also I was working as a volunteer in a Lab with the possibility to have a position in a near future, but now I cannot return to my duties until further notice.” Participant 2

“My productivity this year will directly impact whether I have a competitive K award application.” Participant 3

### Stress and Anxiety

The final theme to emerge that was prevalent across all the previous themes, was that of stress and anxiety. This overarching theme was evident as trainees worried about their own health as well as that of their families, managing their work–life balance, and finding solutions to childcare issues. There was additional stress in trying to adjust to an unfamiliar work-from-home environment and reduced face-to-face contact with colleagues and teammates. Furthermore, anxiety was clearly prevalent in participant responses regarding the impact of COVID-19 on training and future job prospects. Participants directly requested help in extending research time or finding solutions to some of these challenges. In addition, one participant highlighted the wider social context of the Black Lives Matter movement and the social unrest that began in late May as an added source of stress.

“It’s even more stressful than usual to juggle homelife with work at the moment.” Participant 9

“General political and social unrest resulting from this tumultuous time has also made it difficult to focus.” Participant 13

“I’m worried about my health of my family, I’m worried about my career and where I go from here. There’s a lot to be concerned about at the moment.” Participant 2

## Discussion

We found that TL1 trainees and KL2 scholars funded under the auspices of ConduITS, the Institute of Translational Sciences at ISMMS in New York City, in the epicenter of the COVID-19 pandemic, experienced widespread disruption in their research training endeavors as a result of the COVID-19 pandemic. The high-response rate to this survey perhaps indicates how important these issues surrounding pandemic-related disruptions are to the trainees.

This survey revealed that one of the major causes of disruption was the clinical redeployment of both trainees and their mentors, and the loss of protected time for research. For trainees who were not redeployed the “stay-at-home” directives required trainees to adapt to a home environment in which to work, thereby reducing access to team members, clinical and laboratory resources. The shutdown of research studies at ISMMS specifically reduced access to study participants and patient samples need for research progress.

A “new normal” is cautiously resuming in the MSHS as laboratories have gradually reopened, albeit with altered schedules and modified capacity to allow for social distancing practice; IRB-approved patient-oriented research is once again permitted, enabling new trials to be submitted for IRB approval and ongoing trials to gradually reopen to accrual; and protected research time has been reinstated. Yet, there remain legitimate trainee concerns that several months of unprecedented disruption may have a longer term impact on their careers. The survey unveiled considerable uncertainty about future career opportunities and choices due to disruption of job interviews and delayed grant submissions and research publications. This resulted in concern that reduced productivity across these months would have a direct impact on future career options, such as not being prepared to submit for a K award on schedule. Trainees requested extensions to grant funding and ongoing support to complete research projects that were held up, in some cases entirely, for many months. This was the primary concern for many trainees who reported this as a cause of stress and anxiety.

While there are no easy remedies to address these setbacks, we have developed and implemented the following short-term support and developed longer term plans to better enable trainees to remain on track during challenging times. These efforts can be summarized as follows:

### Short-term Support

1. We launched Zoom-based group and one-on-one forums to provide trainees with an opportunity to share their concerns, and delineate individual challenges. Listening, enabling the ability to safely talk through concerns, acknowledging that the pandemic has impacted them greatly, legitimizing trainee concerns, and signposting trainees to appropriate resources is an approach we adopted based upon these findings.
2. We provided one-on-one mentorship to address specific individual actionable needs, accelerate access to needed expertise, and redress specific barriers that individual trainees reported.
3. We leveraged newly established institutional resources to promote well-being and resilience, additionally informed by the impact of pandemic on early stage research careers.
4. We worked with the Mount Sinai Data Warehouse leadership to arrange prioritized TL1 and KL2 trainee access to these resources and personnel in real time, to advance the trainee’s research mission. This included the initiation of video consultations with Data Warehouse and Office of Research Services (ORS) staff to address logistical hurdles and provide guidance and access to know-how.
5. We enhanced the scope of our existing and newly developed online leadership curriculum for early stage TL1 and KL2 investigators to incorporate change management skills and resilience principles. Offering synchronous programs that allow participants to join in real time and asynchronous options that allow participants to learn in their own time.
6. We utilized the Individual Development Planning (IDP) process to enable trainee’s to refocus their career paths and create a plan to get “back on track”; formulate contingency plans and revision of personal and professional goals for research and training; and take into account additional disruptions that may occur in the future during the current pandemic. Mentor-guided revisions to the IDP also enables mentors to more fully realize the impact of COVID-19 on trainee progress, facilitating revisions in and alignment of expectations accordingly.
7. In the short term, we rapidly converted education programs to online platforms to maintain teaching, prevent gaps in the development of essential methodologic skills, and maintain motivation. Moving past this initial phase, these programs were revised and redeveloped with input from instructional designers and online education experts. Additional online education videos and programs are being developed to facilitate training in an online environment and allow trainees to be self-directed in their learning when working from home.
8. We extended the period of funding for our trainees to compensate for the months of research time lost due to the COVID-19 disruption.

### Longer term Plans

In the longer term, the plan at Mount Sinai is to develop an informed institutional strategy to better address the needs of trainees during difficult times, such as the current unanticipated challenge resulting from a global health crisis. To achieve this, we have created a ConduITS-led task force of key stakeholders, which aims to:

1. Leverage ConduITS implemented e-consenting tools that offer a mechanism through which to consent study participants virtually to ensurethe continuation of patient-oriented research efforts.
2. Delineate gaps in needed infrastructure, tools, and processes required to enable TL1 trainees and junior faculty KL2 scholars to overcome recent months of pandemic-related research disruptions in order to rejuvenate and accelerate the progress of their research endeavors and minimize barriers.
3. Expand the recently launched ORS Research Roadmap, an online resource platform to guide and streamline logistics for investigator-initiated patient-oriented research in real time.
4. Expand online asynchronous training opportunities for a wider audience of emerging investigators to advance agility and resilience, building upon an Acceptance and Commitment Therapy Framework. An asynchronous approach allows busy investigators to learn in their own time at their own pace.

Taken together, these specific measures implemented in response to our  survey findings are intended to provide the target support and solutions that our trainees need.

### Limitations

The primary limitation to this study is that the results are from a single site, which limits generalizability in light of different institutions having different approaches to training during the pandemic. However, we hope our reflections and subsequent actions are helpful to other institutions as they think through the “what now” for trainees as new normal resumes.

## Conclusion

Exploring the impact of COVID-19 on trainees at ISMMS in New York City, the initial epicenter of the pandemic in the USA, has provided valuable insight into their experiences, the challenges they have faced, and concerns for their future careers. While challenging, maintaining the education and research mission for our trainees remains a priority as we assist them to overcome the setbacks sustained as a result of the COVID-19 pandemic. While the pain of the pandemic disruption is fresh, it is important that we build contingencies for future research and training operations, as motivation to do so may decrease as normality returns. Lessons learned during COVID-19 pandemic activity in the research training enterprise should inform future planning to lessen strain and disruptions for trainees.
